# Estimating the Combined Effects of Natural and Built Environmental Exposures on Birthweight among Urban Residents in Massachusetts

**DOI:** 10.3390/ijerph17238805

**Published:** 2020-11-27

**Authors:** Maayan Yitshak-Sade, M. Patricia Fabian, Kevin J. Lane, Jaime E. Hart, Joel D. Schwartz, Francine Laden, Peter James, Kelvin C. Fong, Itai Kloog, Antonella Zanobetti

**Affiliations:** 1 Department of Environmental Medicine and Public Health, Icahn School of Medicine at Mount Sinai, New York, NY 10029, USA; 2 Department of Environmental Health, Boston University School of Public Health, Boston, MA 02118, USA; pfabian@bu.edu (M.P.F.); klane@bu.edu (K.J.L.); 3 Harvard T.H. Chan School of Public Health, Boston, MA 02115, USA; rejch@channing.harvard.edu (J.E.H.); joel@hsph.harvard.edu (J.D.S.); Francine.laden@channing.harvard.edu (F.L.); pjames@hsph.harvard.edu (P.J.); kcf502@mail.harvard.edu (K.C.F.); azanobet@hsph.harvard.edu (A.Z.); 4 Channing Division of Network Medicine, Department of Medicine, Brigham and Women’s Hospital and Harvard Medical School, Boston, MA 02115, USA; 5 Department of Population Medicine, Harvard Medical School and Harvard Pilgrim Health Care Institute, Boston, MA 02215, USA; 6 School of the Environment, Yale University, New Haven, MA 06511, USA; 7 Department of Geography and Environmental Development, Faculty of Humanities and Social Sciences, Ben-Gurion University, Beer-Sheva 84105, Israel; ikloog@bgu.ac.il

**Keywords:** birthweight, exposome, air pollution, temperature, noise, greenness

## Abstract

Intrauterine growth has health implications both in childhood and adulthood. Birthweight is partially determined by prenatal environmental exposures. We aim to identify important predictors of birthweight out of a set of environmental, built environment exposures, and socioeconomic environment variables during pregnancy (i.e., fine particulate matter (PM_2.5_), temperature, greenness, walkability, noise, and economic indices). We included all singleton live births of mothers who resided in urban census block-groups and delivered in Massachusetts between 2001 and 2011 (*n* = 640,659). We used an elastic-net model to select important predictors of birthweight and constructed a multivariate model including the selected predictors, with adjustment for confounders. We additionally used a weighted quantile sum regression to assess the contribution of each exposure to differences in birthweight. All exposures were selected as important predictors of birthweight. In the multivariate model, lower birthweight was significantly associated with lower greenness and with higher temperature, walkability, noise, and segregation of the “high income” group. Treating the exposures individually, nighttime noise had the highest weight in its contribution to lower birthweight. In conclusion, after accounting for individual confounders, maternal environmental exposures, built environment exposures, and socioeconomic environment during pregnancy were important predictors of birthweight, emphasizing the role of these exposures in fetal growth and development.

## 1. Introduction

Low birthweight is a strong predictor of perinatal morbidity and mortality [1]. Intrauterine growth has implications on cardiac health, bone development, and other health consequences later in life [2]. Studies suggest that up to 80% of the variation in birthweight is determined by genetic factors [3]. The residual variance is determined partially by maternal health and exposures during pregnancy [4,5,6,7,8,9,10]. Environmental exposures are estimated to contribute about 25% of the variation in birthweight [3]. The contribution of environmental exposures to birthweight reduction might not be large enough to result in a shift from normal to low birthweight (i.e., birthweight < 2500 g. However, given the multiple exposures, and the extent of the population exposed on a daily basis throughout the pregnancy, birthweight differences within the normal range may have meaningful implications on public health on the population level. 

There is extensive literature documenting the association between multiple environmental exposures (e.g., air pollution, temperature, and noise) [11,12,13,14,15] or neighborhood built environment characteristics (e.g., greenness) [16] and birthweight. However, most studies assessed the effect of a single environmental exposure, either without considering other exposures or with the consideration of one additional exposure (usually temperature or fine particulate matter (PM_2.5_)) [17,18]. Given the wide range of environmental exposures that can potentially affect birthweight, it has been challenging to isolate important environmental predictors of birthweight and to identify the exposures that are more highly weighted in their contribution to differences in birthweight. There is a gap in studies that consider the impact of multiple environmental exposures on birthweight while accounting for the correlations between individual and environmental risk factors.

For decades, the research focus of the effects of external environmental exposures has been on the effects of single pollutants. In recent years, the exposome concept has emphasized the need for epidemiological studies that consider many exposures [19]. Only a few recent studies have taken such an exposomic approach when investigating the environmental predictors of birthweight. Hystad et al., for example, have assessed the effect of greenness exposure on birthweight while accounting for multiple environmental exposures (e.g., PM_2.5_, black carbon, NO_2_, noise, walkability, and park proximity) [20]. Another recent study of six European cohorts have estimated associations between low birthweight and multiple exposures defined as the urban exposome including the built environment, air pollution, road traffic noise, meteorology, natural space, and road traffic [21]. Finally, Agier et al. have examined over 100 predictors of birthweight and found lead exposure as a significant risk factor for lower birthweight. After correcting for multiple testing, no other exposure was significantly associated with birthweight [22]. Still, the ability to account for multiple exposures is often limited by sample size and statistical power or by the availability of exposure data. In addition, with multiple spatially and temporally correlated exposures, a priori selection of predictors can be challenging.

In the current study, we apply a combination of variable selection, traditional, and novel regression methods. Using a large cohort of births in Massachusetts, and a wide range of prenatal exposures, we identified important predictors of birthweight out of a set of environmental exposures, built environment characteristics and economic indices, and assessed the relative contribution of each of the selected exposures to differences in birthweight.

## 2. Materials and Methods

### 2.1. Study Population

Our study population were all singleton live births for the years 2001–2011 (*n* = 791,324) extracted from the Massachusetts birth registry data. For each birth, the data included the newborn’s birthweight in grams and sociodemographic and clinical characteristics: date of birth, government support for prenatal care (yes/no—prenatal care paid by the government), race (black/white/other), maternal age at birth, parity (first-born, not first-born), maternal smoking prior to or during pregnancy (yes/no), maternal highest level of education (less than high school, high school, some college, college, advanced degree beyond college), diabetes mellitus (yes/no), chronic hypertension (yes/no), gestational age (in weeks), and the newborn sex. We excluded birthweight < 500 g (*n* = 76) due to higher probabilities of lower birthweight related to high-risk pregnancies and prematurity. We additionally excluded records of gestational age > 42 weeks (*n* = 6362), birthweight over 6 kg (*n* = 133), and records that had missing residence information or missing covariate data (*n* = 116,175) to avoid misclassification due to inaccurate or missing data. Finally, we excluded records of mothers who resided in rural areas (*n* = 38,501) as most of our exposures of interest had little or no variability in non-urban areas. We used the 2010 Census urban and rural classification to define urban/rural areas. This classification is based on population density, and nearby geographic areas containing non-urban land use [23]. In total, we excluded 19% (*n* = 150,665) of all births. The excluded population was similar except for a higher proportion of white women, college graduates, and a higher gestational age at birth (Table S1). For each birth, the maternal residence address was geocoded by the Massachusetts Department of Public Health. This study was approved by the Massachusetts Department of Public Health (approval number 249812) and the human subjects committee at the Harvard T. H. Chan School of Public Health (approval number 20222).

### 2.2. Outcome

Our outcome was birthweight in grams. Birthweight is commonly treated as a marker of the intrauterine environment and is an important predictor of normal growth, healthy development, and survival [2,24].

### 2.3. Exposures

We a priori selected potential environmental exposures based on associations with birthweight in previous studies [7,18,20,25,26]. We examined the following exposures.

1. PM_2.5_: We obtained daily average PM_2.5_ predictions from a model generating predictions at a 1 × 1 km spatial resolution. Out-of-sample “tenfold” cross-validation of the prediction model showed excellent model performance (mean out-of-sample R^2^ = 0.88). For a more in-depth description, refer to Kloog et al. [27].
2. Temperature: We obtained daily average temperature predictions from a model that generated predictions at a 1 × 1 km spatial resolution. Out-of-sample tenfold cross-validation showed excellent model performance (mean out-of-sample R^2^ = 0.94). For more in-depth information refer to Kloog et al. [28].
3. For both the PM and Air temperature modeling we implemented the same rigorous cross validation scheme. We used a “tenfold out of sample” cross-validation scheme to validate our model prediction versus Environmental Protection Agency (EPA) and National Climatic Data Center (NCDC) ground monitoring stations. We randomly divided our data into 90 and 10 percent splits ten times. We predicted values for the 10% data sets using the model fitted from the remaining 90% of the data. To test our results for bias we regressed the measured PM or temperature value for a given site and day against the corresponding predicted value. We estimated the model prediction precision by taking the square root of the mean squared prediction errors (RMSPE). In addition, we calculated prediction errors from a model that contained the spatial components only and calculated temporal R^2^ by regressing Delta measurements of monitoring stations against Delta predicted values where: Delta is the difference between the actual PM/Temperature in place i at time j and the annual mean PM/Temperature at that location, and Delta predicted is defined similarly for the predicted values generated from the model. Spatial R^2^ was calculated by regressing the site-specific annual means in observed PM/Temperature versus the same annual means for predicted PM/Temperature. Models were validated globally but also per region (i.e., Massachussetts) [27,28].
4. Greenness: We used monthly remote sensing data from the MODIS satellites, and averaged the values within each season to calculate the seasonal normalized difference vegetation index (NDVI). NDVI incorporate the plants’ absorption and reflection of light. Its values range from −1.0 to 1.0 with higher values indicate higher levels of vegetative density. Based on previous studies of neighborhood greenness, we used a 250 m buffer around each address to approximate the measure of residential and walking greenness exposure [29,30,31].
5. Walkability index: We used a walkability index which incorporates three census block group level components obtained from the Environmental Protection Agency (EPA) Smart Location Database. [32,33] The index combines z-scores of population density (people/acre) on unprotected land; street intersection density (weighted, with auto-oriented intersections eliminated); and land use diversity based on the mix of retail, office, service, industrial, entertainment, education, healthcare, and public administration employment in the census block group. A higher walkability index indicates a more walkable neighborhood [32].
6. Nighttime noise: We defined median nighttime noise as the exposure of interest. We obtained noise exposure from a non-time varying geospatial sound model that provides nighttime noise level at a 270 m × 270 m spatial resolution [34]. The model incorporates geospatial features (i.e., climate, anthropogenic activity, and empirical acoustical data) sampled at 492 sites across the U.S. between 2000 and 2014. The method utilized random forest, a tree-based machine learning algorithm to predict noise based on geospatial features. The noise model was constructed using a training set containing all available observations, and the fit showed excellent correlation with the empirical data (R^2^ = 0.98). In addition, the model was cross-validated using an exhaustive, leave-one-out process to evaluate the accuracy of national scale projections. Each site was omitted, a new model was constructed, and the model output was tested against the observations for the omitted site. The cross-validation procedure showed a root mean squared error of 4.91 dB. As rare conditions are more prone to error, the main systematic source of error was overestimation of sound levels at the quietest sites and underestimation of sound levels at the loudest sites [34,35].
7. Socioeconomic environment: we used two measures of economic segregation and dissimilarity to assess the neighborhood’s socioeconomic status. We used the formulas published by Krieger et al. [36] to calculate measures of economic segregation and dissimilarity at the block group level. These indices have previously been used to assess how the geographic distribution of economic groups affects human health [37,38].
  a. Economic residential segregation (ERS): measures the uniformity of the proportion of people living in high income households (≥$10,000/year) versus the proportion of people living in low income households (<$25,000/year). The ERS ranges between −1 and 1 and grouped into five categories, where the middle category indicates equal distribution of the two extreme economic groups, positive values closer to 1 indicate higher segregation of the “high income” group, and negative values closer to −1 indicate higher segregation of the “low income” group.
  b. Index of economic dissimilarity (IED): compares the distribution of the proportion of people living in households with high and low income in a smaller area (e.g., block groups) versus the distribution in a larger area (e.g., census tracts) (Table S2).

We linked all the exposures to the residential address at birth. Specifically, we assigned temperature, PM_2.5_, noise, and greenness exposures to each mother based on proximity to the residential address at birth. We assigned walkability and economic segregation and dissimilarity indices to each mother based on the block group of residential address. For walkability, noise and economic segregation and dissimilarity indices, we assumed fixed exposures over time. PM_2.5_, temperature, and greenness predicted exposures did vary with time; we therefore calculated the average exposure for each trimester of pregnancy. 

### 2.4. Statistical Analysis

To assess the relative contribution of multiple environmental exposures during pregnancy on birthweight, we followed several steps. We used Spearman correlation tests to understand the correlations between all exposures. Then, to describe the direction of the relationship between each exposure and birthweight, we fitted separate linear regression models for each exposure.

Before doing so, we assessed the linearity of the relationship between the exposures and birthweight by creating a multivariate model including penalized spline functions for all the exposures. Both the shape dose response curve and the potential biological plausibility were considered in our decision whether to include nonlinear terms. Due to the complexity of the analysis, we tended to include linear terms. Although there is evidence of nonlinear effects between temperature exposure and birth outcomes [4,12,39], we found a linear dose response curve for temperature, and therefore treated the exposure as linear. For ERS, we found a nonlinear relationship, and therefore created a categorical variable as described in the Exposure section. The association with nighttime noise was not linear, showing a protective effect for levels higher than 46 dB. However, as the majority of the noise distribution was lower than 46 dB (80%), and as there is probably no reasonable explanation for the protective effect other than a measurement error, we decided to treat noise exposure as linear. For all other exposures, we used linear terms (Figure S1).

We adjusted each model for the covariates available in the birth records shown in prior studies as potential confounders of the associations tested in our study [9,39]: season and year of birth, government support for prenatal care, race, age at birth, parity, maternal smoking before or during pregnancy, maternal highest level of education attained, diabetes mellitus, chronic hypertension, and gestational age at birth [5,9,29]. We defined the season of birth as follows; Winter—December to February, Spring—March to May, Summer—June to August, and Fall—September to November.

Multiple linear regression is the classical approach to examine the association between maternal or neonatal risk factors and birthweight. However, in cases of multicollinearity (when the exposures are highly correlated) effect estimates obtained from a standard multivariate regression model may be unreliable. In cases of over fitting (where the number of predictors in the model approaches the number of observations in the analyzed population), the variance can be inflated, providing insufficient statistical power to detect small effects. To reduce the impact of collinearity and model overfitting, we used elastic net regression modeling to select the exposures (PM_2.5_, temperature and NDVI in each trimester, walkability, noise, ERS, and IED) to be included in the final multivariate model [40,41,42].

The method applies a weighted penalty to the regression coefficients to achieve asymptotic normality and consistent selection [40,41,42]. In this model, to fully adjust for all potential confounders, we included all the fixed covariates mentioned above, and we applied the penalty to the standardized exposures but not the confounders. We selected the model that minimizes the cross-validation error, with the alpha value that minimized the mean standardized error (0.1), which yields a model result in which some coefficients are shrunk exactly to zero and some are non-zero, and we selected the exposures with non-zero coefficients. As the elastic net model leaves the redundant covariates out of the model, we refer to the selected exposures throughout the manuscript as important predictors of birthweight. 

Finally, we ran a multivariate linear regression model, including all the selected exposures, to obtain the unpenalized direct effect estimates of each exposure on birthweight. We present the results as the difference in birthweight (with 95% confidence intervals (CI) for an IQR rise in exposures).

As a secondary analysis, to assess the relative contribution of each of the selected exposures to birthweight at term, we used a Weighted Quantile Sum (WQS) regression. The WQS regression summarizes all the exposures into one index while taking the association with the outcome into account, and the contribution of each exposure is weighted based on its relevance to the overall association with the outcome. The weights were assigned to quartile-scored exposures within the composite index and were constrained to be between zero and one and summing up to one. Therefore, the exposures with the highest weights are those which contribute most to the outcome, taking the correlation between the exposures into account [43]. We dedicated 60% of the data for training and 40% for validation. As WQS can only incorporate linear predictors, we treated ERS as a confounder, rather than an exposure, for this analysis. As a WQS model requires all the associations to follow a consistent direction, we could only include exposures that are negatively associated with birth weight. For the WQS analysis we therefore transformed the greenness exposures, which were positively associated with birthweight, into new variables where higher values indicate lower greenness.

### 2.5. Sensitivity Analyses

As exposures are clustered within block-groups, we repeated the multivariate regression model adding a random intercept for each block-group of residence. Second, to make sure the associations found in our study are not confounded by socioeconomic status (SES), we repeated the models with adjustment for census block-group median household income and percent poverty. In addition, we have restricted our study population to term-births, to make sure our findings are robust. Finally, we added two WQS sensitivity analyses calculating the weighted contribution of each of the selected exposures to a negative difference in birthweight with weights assigned to tertile-scored and quintile-scored exposures.

## 3. Results

Table 1 shows the maternal and neonatal characteristics of the 640,659 births. The mean birthweight was 3379.3 g, 48.8% of the newborns were female, and the mean gestational age at birth was 39.0 weeks. The mothers were 30.1 years old on average, 69.9% were white, and 33.1% received governmental support.

Figures S2–S8 show the maps with the distributions of the exposures across urban census block-groups. The mean (standard deviation) of the exposures during pregnancy were 10.4 (1.9) µg/m^3^ for PM_2.5_, 11.1 (4.7 °C) for temperature, and 0.5 (0.2) for NDVI. For the walkability index, where higher values indicate neighborhoods that encourage walking, the values ranged between −2.8 and 16.9 and the mean index was 1.1 (1.9). The mean nighttime noise level was 43.5 (2.9 dB). Thirty-nine percent of the mothers resided in block-groups with similar distribution of the high (≥$100 K/year) and low (<$25 K/year) income groups. The mean index of economic dissimilarity was 16.9%, indicating that in general, the distribution of the “high income” and “low income” groups were similar at the block-group and the census tract level (Table 2).

**Figure 1:**
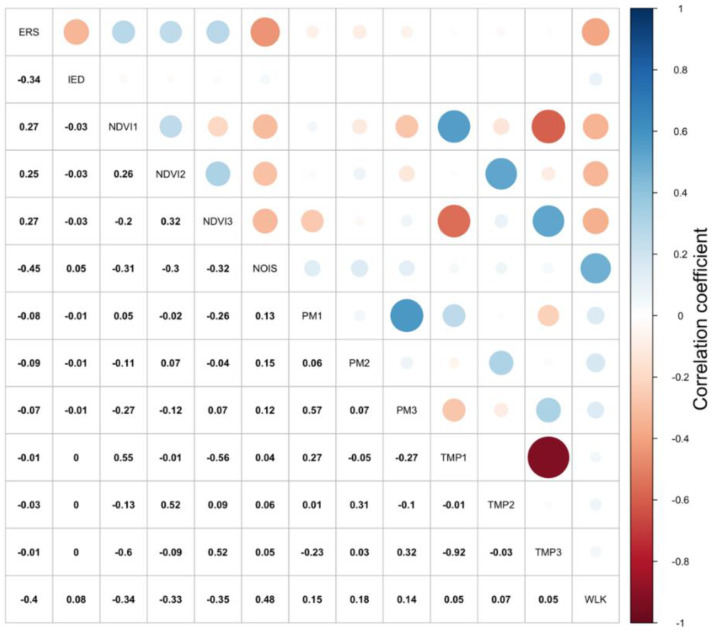
The correlations between the environmental exposures, the built environment characteristics, and the economic indices during pregnancy. Figure 1 shows the correlations between all the exposures. Higher positive correlations are marked with larger blue circles, and higher negative correlations are marked with larger red circles. ERS = economic residential segregation; IED = index of economic dissimilarity; NDVI 1 to 3 = normalized difference vegetation index in the 1st, 2nd, and 3rd trimesters; PM 1 to 3 = fine particulate matter in the 1st, 2nd, and 3rd trimesters; TMP 1 to 3 = temperature in the 1st, 2nd, and 3rd trimesters.

We observed large negative correlations between the average temperature in the first and third trimesters (r = −0.9) and correlations around 0.5 between PM_2.5_ exposure in the first and third trimester and between temperature and NDVI exposures (Figure 1). 

Table 3 shows the multivariable adjusted difference in birthweight for an IQR rise in each exposure, obtained from single exposure models. We observed lower birthweight associated with higher PM_2.5_ exposure in the three trimesters of pregnancy; temperature exposure in the 2nd and 3rd trimester; and with higher walkability, noise, and IED. Conversely, we found that higher greenspace in the three trimesters of pregnancy and residing in block groups with more residents of the ‘high income’ group were associated with higher birthweight.

In the multivariate model including all exposures, temperature exposure in all three trimesters was significantly associated with birthweight. Although attenuated due to adjustment to the other covariates, the associations with noise, ERS, walkability, and NDVI in the 1st and 3rd trimesters remained significantly associated with birthweight. IED, NDVI in the 2nd trimester, and PM_2.5_ were no longer associated with birthweight (Table 3). The associations with the full set of model covariates are presented in Table S3.

All the effect estimates for the associations with birthweight were obtained from linear regressions. The single exposure models tested the association with each exposure separately, with adjustment for maternal and neonatal covariates. The multivariate model includes all the exposures selected by the elastic net model, with adjustment for maternal and neonatal covariates. Each model was adjusted for the following covariates: season and year of birth, government support for prenatal care, the maternal highest level of education attained, race, maternal age, parity, maternal smoking before or during pregnancy, diabetes mellitus, chronic hypertension, and gestational age at birth. We present the results as the difference in birthweight (in grams) for an interquartile (IQR increase in the exposures).

We conducted several sensitivity analyses to make sure the associations found in our study were robust. The addition of random effects to the multivariate regression yielded larger standard errors, as expected, but the significance and the magnitude of the effects remained similar for all the exposures except for temperature. With the inclusion of a random intercept, temperature in the first and second trimesters was no longer significantly associated with birthweight (Table S4a). The inclusion of neighborhood SES adjustment (Table S4b) and the restriction of the study population to term births (Table S4c) did not change the results.

**Figure 2:**
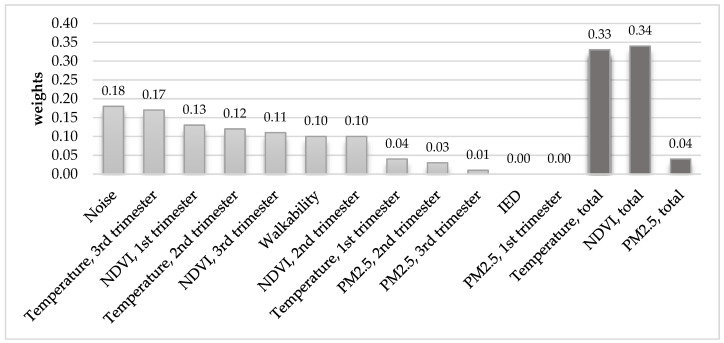
The weighted contribution of each of the selected exposures to a negative difference in birthweight: results of a Weighted Quantile Sum (WQS) regression.

Figure 2 shows results from the WQS showing the weighted contribution associated with rises in each of the exposures and birthweight.

Figure 2 shows the weighted contribution of each exposure to a negative difference in birth weight. Results were obtained from a WQS regression. The WQS regression summarizes all the exposures into one index while taking the association with the outcome into account, and the contribution of each exposure is weighted based on its relevance to the overall association with the outcome. The weights were assigned to quartile-scored exposures within the composite index and were constrained to be between zero and one and summing up to one.

The combined index of exposures was significantly associated with lower birthweight. We found nighttime noise to be the largest contributor to lower birthweight, accounting for 18% of the weights. This result was consistent across the sensitivity analyses using different quantiles. Temperature in the third trimester was equally weighted to noise, but results differed in the sensitivity analyses. IED, temperature in the first trimester, and PM_2.5_ exposures across pregnancy had the smallest contribution to lower birthweight. The relative contribution of NDVI exposures largely differed between the analyses. However, when summing all three trimesters of exposure, NDVI accounted for 34% of the wights in the main analysis and 40% of the weights in the sensitivity analyses (Figures S9 and S10).

## 4. Discussion

We found environmental exposures, built environment exposures, and economic indices during pregnancy to be important predictors of birthweight, even after accounting for known individual maternal and neonatal risk factors. We observed lower birthweights in infants born to women with higher temperature exposures during pregnancy, living in areas with less greenness and higher noise, living in more walkable areas, and in areas with more of the “low income” population. Treating the exposures individually, nighttime noise had the highest weight in its contribution to lower birthweight (graphical abstract). Summing trimestral exposures together, NDVI was the most highly weighed exposure.

Our findings are similar to previous studies where associations with birthweight were observed with maternal exposure to various environmental exposures, among them PM_2.5_ [11,17,25,44,45], temperature [18,46,47], noise [6,7], greenness [5,9], and walkability [20]. Although, most of these studies have assessed the effect of a single exposure at a time, some did try to distinguish between these spatially correlated exposures and studied the effects of multiple exposures simultaneously. For example, Smith et al. found an increased risk for low birthweight associated with PM_2.5_ exposure, independent of road traffic noise. Traffic noise, however, was no longer associated with the outcome once PM_2.5_ was controlled for [6]. Hystad et al. explored the pathways related to the protective effect of greenness on birthweight and found that the beneficial effect of greenness was robust to adjustment for PM_2.5_, black carbon, NO_2_, noise, walkability, and park proximity [20].

Although we observed significant associations of PM_2.5_ with birthweight in the single exposure models, the effects were attenuated in the multi-exposure model. Moreover, although PM_2.5_ exposures were selected by the elastic net, the effect sizes were very small. It is possible that our analysis was underpowered to detect a significant effect of PM_2.5_ exposure in a multivariate model, while adjusting for other environmental exposures. Similar to our findings, Hystad et al. found no association between PM_2.5_ and birthweight when including NDVI in the model [20]. Nieuwenhuijsen et al., however, did find a significant association between PM_2.5_ and birthweight when including other urban exposome exposures in the model. Like our study, which identified neighborhood greenness as one of the most important predictors of differences in birthweight, this study also found the most consistent statistically significant associations between green space exposure and birthweight [21].

Low socioeconomic status (SES) has been linked to poor obstetric outcomes in many studies [48,49,50]. Most studies that assessed the effect of segregation on birth outcomes focused mainly on the effect of racial segregation in the neighborhood rather than economic segregation [51,52,53]. One study, by Vinikoor et al., found that being economically privileged is protective against low birthweight only in predominantly black neighborhoods. The Vinikoor study, however, used a different economic segregation measure and assessed the effect of maternal residence in census tracts with a higher household income than would be expected based on the mother’s education and marital status [54]. Here, we show that residing in a census block-groups with “low income” economic segregation (i.e., high proportion of people living in low income versus high income households) poses a risk for low birthweight deliveries, independent of the mother’s race. Economic dissimilarity was not associated with birthweight in the multivariate model.

Unlike the established association between birthweight and exposure to air pollution and SES, evidence on noise, greenness, walkability, and temperature exposures is scarce and results are inconsistent. We found birthweight to be inversely associated with temperature and nighttime noise and positively associated with greenness. Most previous studies found birthweight decreases associated with hot temperatures [18,46], some with cold temperatures [55], and others observed no association at all [8,56]. A recent systematic review found evidence for an association between noise exposure during pregnancy and lower birthweight; however, it concluded that most current studies are of low quality and require better exposure assessment and adjustment for SES and environmental confounders [7].

Several studies have demonstrated health benefits of residence in greener neighborhoods [57]. Although a recent review of residential greenness and birth outcomes found consistent positive associations between neighborhood greenness and birthweight, the associations were weak [16]. We found increases in birthweight associated with greenness exposure in the first and third trimesters of pregnancy, among women who reside in urban neighborhoods. We also found lower birthweight associated with higher walkability. Similar to our findings, a study in Connecticut found that urbanicity was negatively associated with birthweight [5].

The exposures tested in our study can affect birthweight through different mechanisms. Exposure to high temperatures during early stages of pregnancy may cause a reduction in placental weight and umbilical cord flow [4]. High-temperature exposures during the final stages of pregnancy may also affect birthweight, but the mechanism for this association is yet to be clear [4]. Exposure to higher air pollution levels may cause oxidative stress, impairing the transport of oxygen and nutrient to the fetus and affect the intrauterine growth [58,59]. Exposure to high noise levels can alter the mother’s levels of stress hormones, reduce ovarian and uterine blood flow and inhibit fetal growth [60]. Residence in a greener neighborhood can have a beneficial effect on fetal growth through mitigation of harmful environmental exposures (i.e., filter air pollution and provide shade), or through the opportunity for social interactions and physical activity, contributing to better maternal health and lower stress levels [9]. Higher neighborhood walkability can be associated with higher physical activity and therefore a healthier maternal lifestyle and higher birthweight. On the one hand, it can be associated with higher urbanicity, traffic, and pollution levels, and therefore associated with lower birthweight [20]. To the best of our knowledge, there are no studies that assessed the effect of economic residential segregation on birthweight. However, there is a study that examined the effect of racial segregation on birthweight among black mothers, which found a higher probability of low birthweight in more segregated metropolitan areas [61]. Residence in neighborhoods with lower SES can increase the risk for lower birthweight through limited access to housing, healthcare services, healthy nutrition, quality education and more [48,49]. Similarly, we hypothesize that residence in block groups with higher economic segregation, similar to racial segregation, can have adverse effects on birthweight due to higher levels of poverty and crime, higher exposure to stress, fewer options for purchasing healthy food, and limited access to health care [61,62].

The major strength of our study is the inclusion of a large sample of births, and a wide range of environmental predictors of birthweight. Another strength is the combination of two statistical approaches. The elastic net regression, which allowed us to select important predictors of birthweight while accounting for the complex correlations between these multiple exposures.

The analysis of health outcomes that are affected by a complex net of exposures requires tools that will help distinguish the contribution and weight of each exposure while accounting for other exposures. As the elastic net model has the ability to distinguish between the redundant exposures, and to identify exposures that are important predictors of birthweight, this method is an important tool in environmental research, where health outcomes are affected by multiple exposures, and where exposures are often correlated. Within the pool of important predictors, we also used WQS regression, and identified the exposures that had higher weight in their contribution to a negative difference in birthweight. These results can be of use to expecting families in their choice of place of residence. No place of residence is free of environmental exposures, and there is always a tradeoff of exposures. This tool, however, can provide insights on exposures that contribute more to differences in birthweight, and help focus the efforts to reduce exposure to nighttime noise and temperature, and increase exposure to greenness.

Our study had several limitations. First, we excluded 12% of the records due to missing data. However, this problem is common in studies that use routinely collected data. In addition, as the excluded population was mostly similar to the population included in the analysis, we do not expect it to cause a selection bias. Second, as we assigned exposure based on maternal residence reported at the time of birth, we might have had misclassified exposure for women who changed addresses as conception. It is unlikely that women based their decision regarding the new place of residence on air pollution or temperature exposure. Additionally, it is unlikely that a pregnancy will result in a move to a new residence with a very different socioeconomic environment. We therefore expect the measurement error for these exposures to be non-differential and therefore we do not expect it to bias our results. It is possible, however, that pregnant women will relocate to quieter, greener, and/or less walkable areas. Therefore, for these exposures, the measurement error could potentially bias the results toward the null. Third, we did not have information on other maternal factors that may confound the association between the tested exposures and birth weight. Maternal weight for example was reported to be associated with birthweight [63], as well as with air pollution, neighborhood greenness, and noise exposure [64,65]. Factors such as genetic susceptibility [3] and substance-using mothers [66], although related to birthweight, are not likely to be related to environmental exposures and therefore are not likely to confound the associations in our study. Finally, we could not evaluate trimester-specific exposures to walkability and noise, which were only calculated once during the study period. However, as these land use characteristics likely vary very slowly over time, we do not expect it to bias our results.

In addition to the aforementioned limitations, our study is subjected to the limitations of the methods used in our analysis. The elastic net model selects variables with non-zero coefficients, regardless of the effect size and clinical meaning of this effect. In addition, although the elastic net model is capable of identifying important predictors of birthweight, it may have a tendency to incorrectly select a large number of predictors in cases of strong associations with the outcome [67]. However, as we estimated small environmental effect sizes, we do not expect this limitation to affect the variable selection process in our analysis. Lastly, a multi-phase approach in a statistical analysis may result in model overfitting. However, as the aim of the first stage in our statistical approach (i.e., single exposure models) was descriptive and was not used to select variables, this stage was not a part of the selection process and therefore unlikely to result in overfitting. Furthermore, as the elastic net model found all the covariates as predictors of the outcome, overfitting of the final multivariate regression, which included the exact same covariates, is unlikely in our analysis.

A major limitation of the WQS regression is that it can only include variables for which the effect on the outcome is in the same direction. We therefore could not include economic residential segregation as an exposure in this analysis. In addition, the WQS regression may lose information on the continuous range of exposure due to the use of quartiles. We therefore added sensitivity analyses, assigning different quantiles to exposures to create the weights. Although the results differed depending on the quantiles used, nighttime noise was consistently the most highly weighted exposure. Last, WQS requires strong assumptions of linear effects and lack of interactions between exposures [43].

## 5. Conclusions

In conclusion, even after accounting for maternal and neonatal characteristics, we found multiple environmental exposures, built environment exposures, and neighborhood economic indices during pregnancy to be associated with differences in birthweight, emphasizing the complex role of multiple exposures in fetal growth and development. The inclusion of multiple exposures in the model provides avenues for intervention across different exposure dimensions.

## Figures and Tables

**Table 1:** Maternal and neonatal characteristics of women who gave birth in Massachusetts between 2001 and 2011.

*N*	640,659
Maternal characteristics	
Age, mean ± SD	30.1 (6.8)
Race, *n* (%)	
White	446,053 (69.9%)
Black	53,359 (8.5%)
Other	141,247 (21.6%)
Education, *n* (%)	
Less than high school	72,606 (11.3%)
High school	161,774 (25.3%)
Some college	137,743 (21.5%)
College	166,484 (26.0%)
More than college	102,052 (15.9%)
Government support	213,462 (33.3%)
Smoking, *n* (%)	91,050 (14.2%)
Parity > 1, *n* (%)	292,604 (54.7%)
Diabetes mellitus, *n* (%)	6095 (1.0%)
Chronic hypertension, *n* (%)	8180 (1.3%)
Neonatal characteristics	
Birthweight, mean ± SD (g)	3379.3 ± 533.2
Sex, *n* (%)	
Female	312,706 (48.8%)
Male	327,953 (51.2%)
Gestational age, mean ± SD	39.0 ± 1.6
Census-block SES characteristics	
Percent poverty	2.5% ± 3.2%
Median household income	70,627 ± 35,693

Table 1 shows the maternal, neonatal, and census block group socioeconomic characteristics of the study population. Maternal diabetes and hypertension refer to chronic conditions, not induced by gestation. Gestational age at birth is calculated from the last menstrual period.

**Table 2:** Summary statistics of the environmental exposures, built environment characteristics, and economic indices during pregnancy of women who gave birth in Massachusetts between 2001 and 2011 (*n* = 640,695).

Exposure	Summary Statistics	Range (Min; Max)	25th Percentile	50th Percentile	75th Percentile
PM2.5 (µg/m3, Mean ± SD)					
1st trimester	10.5 ± 1.9	(3.9; 22.9)	9.0	10.3	11.8
2nd trimester	10.4 ± 1.7	(4.3; 20.5)	9.1	10.2	11.5
3rd trimester	10.4 ± 2.0	(3.9; 23.3)	8.8	10.2	11.7
Temperature (°C, Mean ± SD)					
1st trimester	10.9 ± 4.8	(−1.6; 29.7)	6.5	10.8	15.4
2nd trimester	11.2 ± 4.5	(−1.2; 33.2)	7.1	11.2	15.4
3rd trimester	11.4 ± 4.8	(−4.1; 29.6)	7.1	11.7	15.8
Greenness (NDVI, Mean ± SD)					
1st trimester	0.5 ± 0.2	(−0.2; 0.9)	0.3	0.5	0.7
2nd trimester	0.5 ± 0.2	(−0.2; 0.9)	0.3	0.5	0.7
3rd trimester	0.5 ± 0.2	(−0.2; 0.9)	0.3	0.5	0.6
Walkability,( Mean ± SD)	1.1 ± 1.9	(−2.8; 16.9)	−0.1	0.7	2.0
ERS, *n* (%)					
−1 to −0.6	16,729 (2.6)				
−0.6 to −0.2	121,562 (19.0)				
−0.2 to 0.2	251,704 (39.3)				
0.2 to 0.6	214,642 (33.5)				
More than 0.6	36,022 (5.6)				
IED (%, Mean ± SD)	16.9 ± 11.2	(0.0; 51.3)	8.4	14.8	23.3
Median Nighttime Noise (dB, Mean ± SD)	43.5 ± 2.9	(29.7; 53.5)	41.5	42.9	45.6

**Table 3:** The association between birthweight and each environmental exposure, built environment exposure, and economic indices during pregnancy in single, and multi-exposure models (*n* = 640,695).

	IQR	Difference in Weight (g per IQR Increase (95% CI))	
Exposure		Single Exposure Models	Multi-Exposure Models
PM_2.5_, 1st trimester (µg/m^3^)	2.8	−5.4 (−7.53; −3.28) *	0.96 (−1.65; 3.56)
PM_2.5_, 2nd trimester (µg/m^3^)	2.4	−9.4 (−11.59; −7.21) *	−0.83 (−3.34; 1.69)
PM_2.5_, 3rd trimester (µg/m^3^)	2.8	−7.76 (−10.07; −5.46) *	−0.44 (−3.16; 2.29)
Temperature, 1st trimester (°C)	8.8	0.59 (−3.3; 4.48)	−10.27 (−17.68; −2.87) *
Temperature, 2nd trimester (°C)	8.3	−10.6 (−14.58; −6.62) *	−4.85 (−9.08; −0.63) *
Temperature, 3rd trimester (°C)	8.6	−15.75 (−19.84; −11.66) *	−19.3 (−27.04; −11.57) *
NDVI, 1st trimester	0.3	20.89 (18.55; 23.23) *	5.71 (2.97; 8.45) *
NDVI, 2nd trimester	0.3	17.94 (15.7; 20.19) *	2.53 (−0.17; 5.23)
NDVI, 3rd trimester	0.3	20.03 (17.79; 22.26) *	5.87 (3.22; 8.53) *
Walkability	2.2	−5.4 (−7.53; −3.28) *	−5.63 (−7.24; −4.02) *
ERS			
−1 to −0.6		Reference	Reference
−0.6 to −0.2		21.39 (14.28; 28.5) *	15.99 (8.85; 23.14) *
−0.2 to 0.2		42.68 (35.68; 49.67) *	28.64 (21.52; 35.77) *
0.2 to 0.6		53.39 (46.19; 60.6) *	32.12 (24.61; 39.63) *
0.6+		44.88 (36.48; 53.27) *	19.46 (10.58; 28.34) *
IED	14.9	−2.14 (−3.58; −0.71) *	−0.32 (−1.83; 1.18)
Noise levels, (dB)	4.1	−16.88 (−18.49; −15.27) *	−5.63 (−7.52; −3.73) *

ERS = Economic Residential Segregation; IED = Index of Economic Dissimilarity; NDVI = normalized difference vegetation index. * *p* < 0.05.

## Supplementary Materials

The following are available online at https://www.mdpi.com/1660-4601/17/23/8805/s1, Table S1: Maternal and neonatal characteristics of women who were excluded from the analysis. Table S2: Census area economic segregation and dissimilarity indices. Table S3: A multi-exposure model for the association between birthweight and each environmental exposure, built environment characteristic and economic indices during pregnancy. Table S4: Sensitivity analyses: the association between birthweight and each environmental exposure, built environment characteristic, and economic indexes during pregnancy in a multi-exposure model. Figure S1: The associations between the exposures and birthweight using penalized splines to allow for nonlinear associations: results of a multivariate regression. Figure S2: The mean PM_2.5_ exposure during pregnancy per census block group, among women who gave birth in Massachusetts between 2001 and 2011. Figure S3: The mean temperature exposure during pregnancy per census block group, among women who gave birth in Massachusetts between 2001 and 2011. Figure S4: The mean greenness exposure during pregnancy per census block group, among women who gave birth in Massachusetts between 2001 and 2011. Figure S5: The mean noise exposure during pregnancy per census block group, among women who gave birth in Massachusetts between 2001 and 2011. Figure S6: The mean walkability index during pregnancy per census block group, among women who gave birth in Massachusetts between 2001 and 2011. Figure S7: The mean economic residential segregation (ERS) exposure during pregnancy per census block group, among women who gave birth in Massachusetts between 2001 and 2011. Figure S8: The mean index of economic dissimilarity (IED) exposure during pregnancy per census block group, among women who gave birth in Massachusetts between 2001 and 2011. Figure S9: The weighted contribution of each of the selected exposures to a negative difference in birthweight: results of a Weighted Quantile Sum (WQS) regression with weights assigned to tertile-scored exposures. Figure S10: The weighted contribution of each of the selected exposures to a negative difference in birthweight: results of a Weighted Quantile Sum (WQS) regression with weights assigned to quintile-scored exposures.
