# Supplementary material for: Estimating the Combined Effects of Natural and Built Environmental Exposures on Birthweight among Urban Residents in Massachusetts
Source: Int J Environ Res Public Health. 2020 Nov 27;17(23):8805. doi: 10.3390/ijerph17238805 (PMC7731163; doi:10.3390/ijerph17238805)
Supplement: Supplementary file 1 [file ijerph-17-08805-s001.pdf]

Supplemental Material

Table S1. Maternal and neonatal characteristics of women who were excluded from the analysis.

N	417,843
Maternal	
Age, mean ± SD	30.3 (5.9)
Race, *n* (%)	
White	313,248 (75.6)
Black	41,503 (10.0)
Other	63,092 (14.4)
Education, *n* (%)	
Less than high school	42,762 (10.3)
High school	90,880 (21.9)
Some college	100,421 (24.2)
College	108,792 (26.2)
More than college	71,685 (17.3)
Government support	133,137 (32.3)
Smoking, *n* (%)	54,556 (13.1)
Parity > 1, *n* (%)	159,599 (43.2)
Diabetes mellitus, *n* (%)	4,262 (1.0)
Chronic hypertension, *n* (%)	6,171 (1.5)
Neonatal	
Birthweight, mean ± SD	3251.5 ± 708.3
Female sex, n (%)	204,352 (48.9)
Gestational age, mean ± SD	41.9 ± 14.3
Census block SES	
Percent poverty	2.0% ± 3.0%
Median household income	7381.2 ± 35718.2

Table S2. Census area economic segregation and dissimilarity indices (indices were from Krieger 2017 [26]).

Measure	Formula	Possible values	Interpretation
Economic Residential Segregation (ERS) index	$ERS= ((N_{hincome \geq \$100,000}) - (N_{hincome < \$25,000}))/ N_{total}$<br>Where *Nhincome ≥$100,000* is the number of persons in households with income ≥$100,000; *Nhincome <$25,000* is the number of persons people in households with income <$25,000, and *Ntotal* is the total population with household income data in each census block group.	Range from −1 to 1.	Values approaching -1 indicate a higher proportion of persons living in low income households, a value of 0 indicates equal proportion of persons living in high and low income households, and values approaching 1 mean a higher proportion of persons living in high income households.
Index of Economic Dissimilarity (IED)	$IED = (0.5 \Sigma \lvert (B_{hincome \geq \$100,000} / T_{hincome \geq \$100,000}) - B_{hincome < \$25,000} / T_{hincome < \$25,000}) \rvert) * 100$<br>Where *Bhincome ≥$100,000* is the number of persons living in households with income ≥$100,000 in a block group; *Thincome ≥$100,000* is the number of persons living in households with income ≥$100,000 in a census tract; *Bhincome <$25,000* is the number of persons living in households with income < $25,000 in a block group and *Thincome > $25,000* is the number of persons living in households with income < $25,000 in a tract.	Range from 0% to 100%,	High values indicate high dissimilarity in the distribution of low and high income groups between the block group and the Census tract, and low values indicate similarity.

Krieger N, Waterman PD, Batra N, Murphy J, Dooley D, Shah S. 2017. Measures of local segregation for monitoring health inequities by local health departments AJPH Methods 107 903–906.

Table S3. A multi-exposure model for the association between birthweight and each environmental exposure, built environment characteristic and economic indices during pregnancy.

Variable	Difference in birth weight	95% Confidence intervals		*P* value
Season (reference: winter)				
Spring	12.14	6.37	17.91	<0.001
Summer	12.06	4.66	19.45	0.0014
Fall	7.93	2	13.86	0.0088
Maternal age, years	1.54	1.31	1.76	<0.001
Parity (2 or more)	-127	-129.32	-124.68	<0.001
Smoking	-82.49	-85.78	-79.21	<0.001
Diabetes Mellitus	177.93	166.82	189.03	<0.001
Hypertension	-65.13	-74.75	-55.5	<0.001
Gestational age, weeks	174.07	173.41	174.73	<0.001
Maternal race (reference: white)				
Black	-115.13	-119.27	-111	<0.001
Other	-99.47	-102.4	-96.55	<0.001
Year of birth (reference: 2001)				
2002	-2.69	-8.81	3.42	0.3876
2003	2.18	-3.77	8.12	0.4729
2004	4.36	-1.93	10.64	0.1740
2005	-4.62	-10.63	1.39	0.1320
2006	-6.65	-12.99	-0.32	0.0396
2007	-4.00	-10.69	2.69	0.2410
2008	1.51	-4.63	7.65	0.6297
2009	-4.32	-11.27	2.62	0.2226
2010	-8.65	-17.01	-0.3	0.0424
2011	14.92	5.51	24.33	0.0019
Education (reference: less than high school)				
High school	14.81	10.83	18.79	<0.001
Some college	48.16	43.82	52.49	<0.001
College	44.71	40	49.42	<0.001
More than college	33.86	28.72	39	<0.001
Government support	-27.87	-30.85	-24.89	<0.001
$PM_{2.5}$, 1st trimester (μg/m3)	0.34	-0.59	1.28	0.4710
$PM_{2.5}$, 2nd trimester (μg/m3)	-0.34	-1.39	0.7	0.5198
$PM_{2.5}$, 3rd trimester (μg/m3)	-0.15	-1.11	0.81	0.7542
Temperature, 1st trimester (^{0}C)	-1.16	-2	-0.32	0.0066
Temperature, 2nd trimester (^{0}C)	-0.58	-1.09	-0.08	0.0243
Temperature, 3rd trimester (^{0}C)	-2.22	-3.11	-1.33	<0.001
NDVI, 1st trimester	15.65	8.13	23.17	<0.001
NDVI, 2nd trimester	6.94	-0.47	14.34	0.0664
NDVI, 3rd trimester	17.54	9.6	25.47	<0.001
Walkability	-2.56	-3.29	-1.83	<0.001
Economic Residential Segregation (reference: -1 to -0.6)				
-0.6 to -0.2	15.99	8.85	23.14	<0.001
-0.2 to 0.2	28.64	21.52	35.77	<0.001
0.2 to 0.6	32.12	24.61	39.63	<0.001
0.6+	19.46	10.58	28.34	<0.001
Index of Economic Dissimilarity	-0.02	-0.12	0.08	0.6727
Noise levels, (dB)	-1.36	-1.82	-0.91	<0.001

We present the results as the difference in birthweight (in grams) for one unit increase in the exposures.

Table S4. Sensitivity analyses: the association between birthweight and each environmental exposure, built environment characteristic, and economic indexes during pregnancy in a multi-exposure model.

	Change in weight (g) per IQR increase (95% CI)		
**Exposure**	**(a)**	**(b)**	**(c)**

$PM_{2.5}$, 1st trimester (µg/m3)	1.4 (-1.3; 4.0)	0.9 (-1.7; 3.5)	-0.3(-2.9; 2.4)
$PM_{2.5}$, 2nd trimester (µg/m3)	-0.1 (-2.7; 2.5)	-0.8 (-3.3; 1.7)	-1.4(-3.9; 1.2)
$PM_{2.5}$, 3rd trimester (µg/m3)	-0.3 (-3.1; 2.4)	-0.5 (-3.2; 2.2)	0.4(-2.4; 3.2)
Temperature, 1st trimester (0C)	-3.7 (-11.7; 4.4)	-9.8 (-17.2; -2.4) *	-16.3(-24.1; -8.4) *
Temperature, 2nd trimester (0C)	-3.8 (-8.1; 0.3)	-4.7 (-9.0; -0.5) *	-6.9(-11.3; -2.5) *
Temperature, 3rd trimester (0C)	-12.4 (-20.6; -3.7) *	-18.6 (-26.3; -10.8) *	-20.7(-28.8; -12.6) *
NDVI, 1st trimester	4.5 (1.6; 7.3) *	5.8 (3.1; 8.6) *	5.3(2.5; 8.1) *
NDVI, 2nd trimester	1.8 (-0.9; 4.6)	2.6 (-0.1; 5.3)	4.2(1.4; 6.9) *
NDVI, 3rd trimester	4.8 (2.1; 7.6) *	5.9 (3.1; 8.6) *	4.2(1.5; 6.9) *
Walkability	-7.2 (-9.3; -5.1) *	-5.6 (-7.2; -4.0) *	-4.3(-5.9; -2.6) *
Economic Residential Segregation (ERS)			
-1 to -0.6	Reference	Reference	Reference
-0.6 to -0.2	14.5 (4.3; 24.7) *	11.7 (4.2; 19.2) *	15.5(8.2; 22.9) *
-0.2 to 0.2	28.0 (17.9; 38.1) *	22.7 (14.5; 30.9) *	28.6(21.3; 35.9) *
0.2 to 0.6	32.0 (21.6; 42.5) *	28.1 (18.7; 37.4) *	31.7(23.9; 39.4) *
0.6+	17.3 (5.3; 29.4) *	20.1 (8.9; 32.9) *	16.7(7.6; 25.8) *
Index of Economic Dissimilarity (IED)	-0.4 (-2.5; 1.7)	-0.2 (-1.7; 1.3)	-0.7(-2.2; 0.8)
Noise levels, (dB)	-7.1 (-9.6; -4.6) *	-5.4 (-7.3; -3.5) *	-5.8(-7.7; -3.8) *

(a) with a random intercept for each census block-group; (b) with adjustment for maternal census block-group median household income and percent poverty; (c) restricted only to term births (n = 601,927 births). * $p < 0.05$.

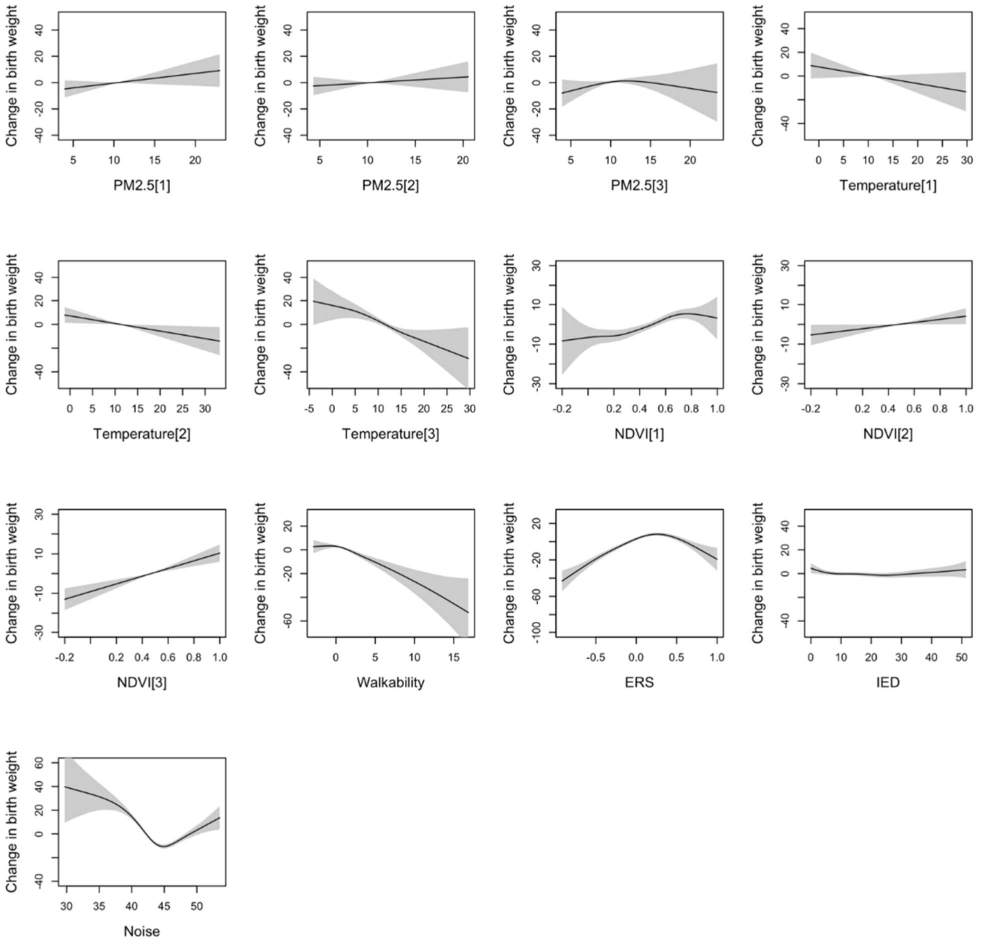

Figure S1. The associations between the exposures and birthweight using penalized splines to allow for nonlinear associations: results of a multivariate regression.

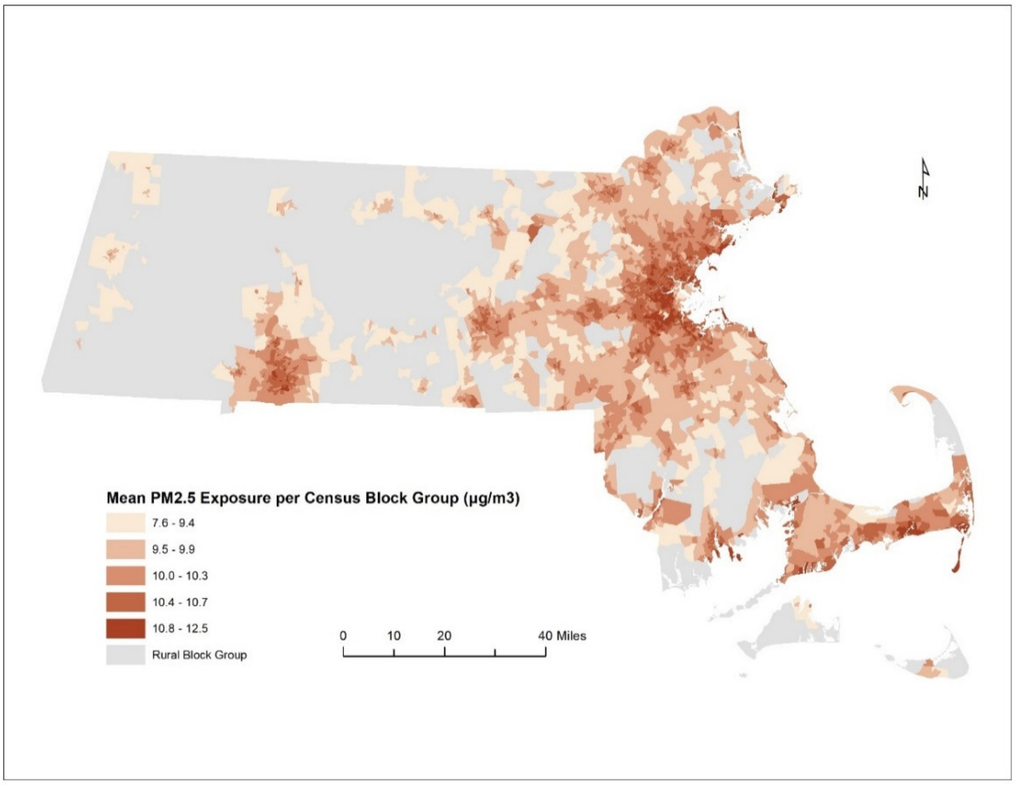

Figure S2. The mean $PM_{2.5}$ exposure during pregnancy per census block group, among women who gave birth in Massachusetts between 2001 and 2011.

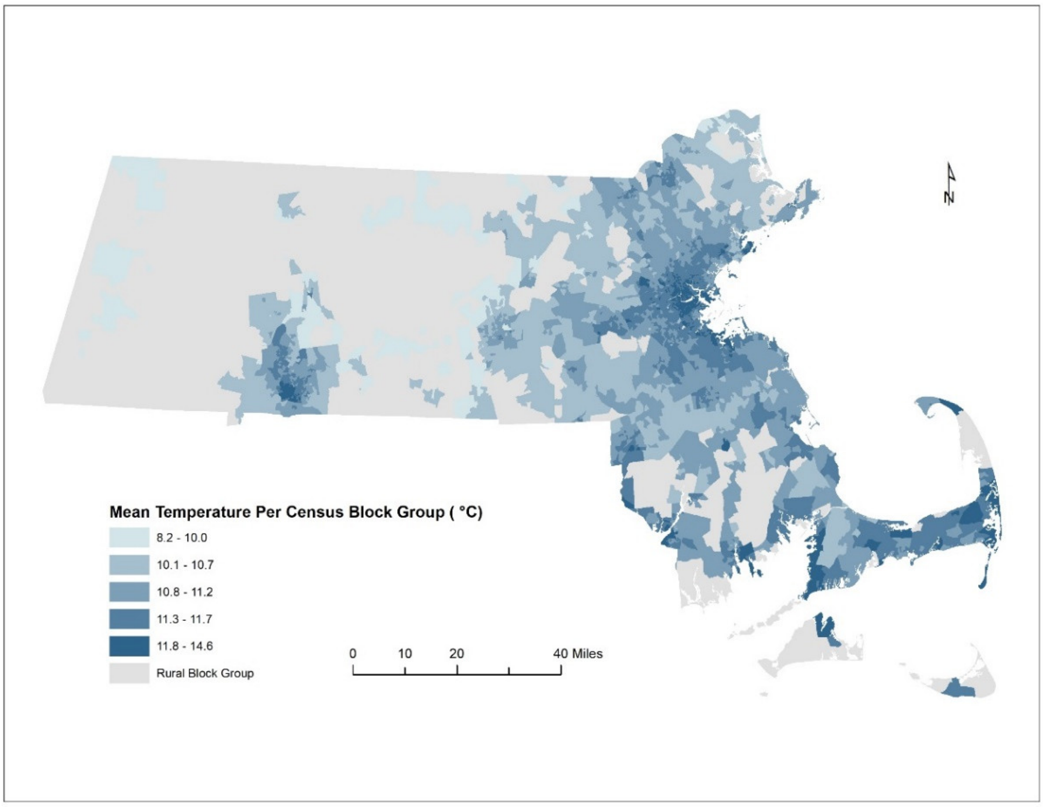

Figure S3. The mean temperature exposure during pregnancy per census block group, among women who gave birth in Massachusetts between 2001 and 2011.

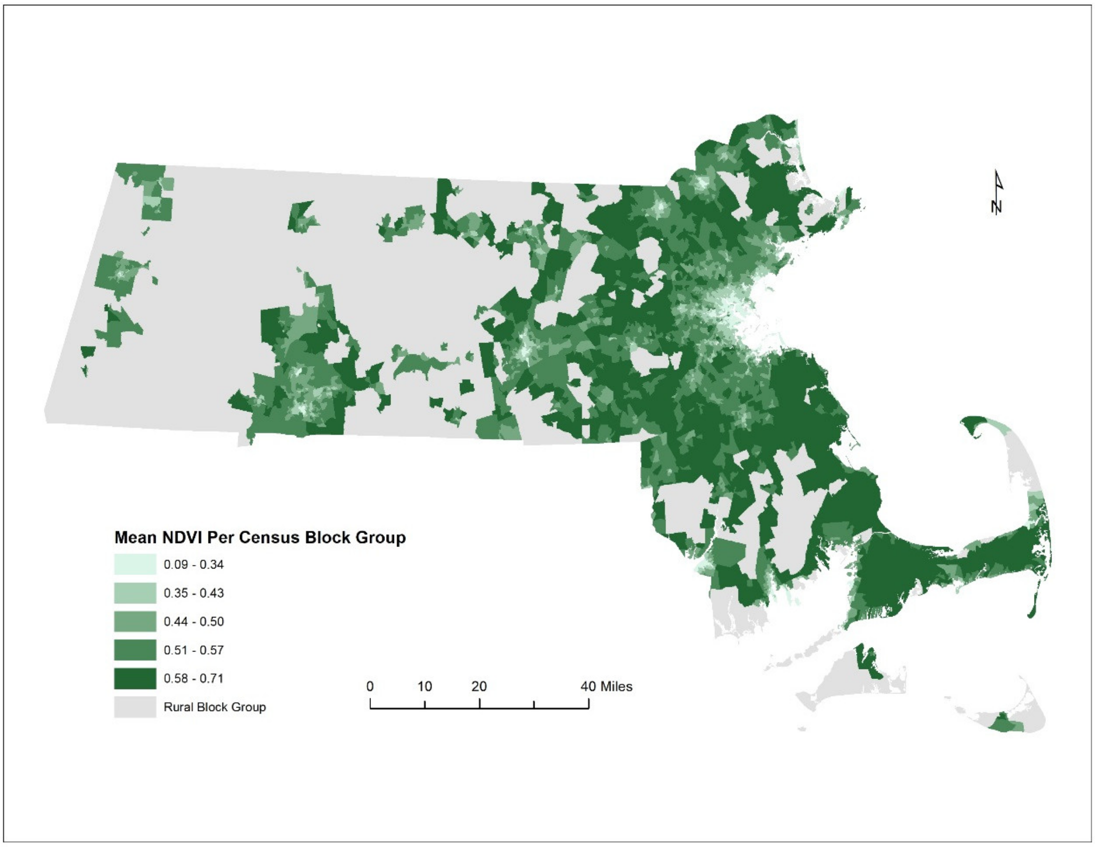

Figure S4. The mean greenness exposure during pregnancy per census block group, among women who gave birth in Massachusetts between 2001 and 2011.

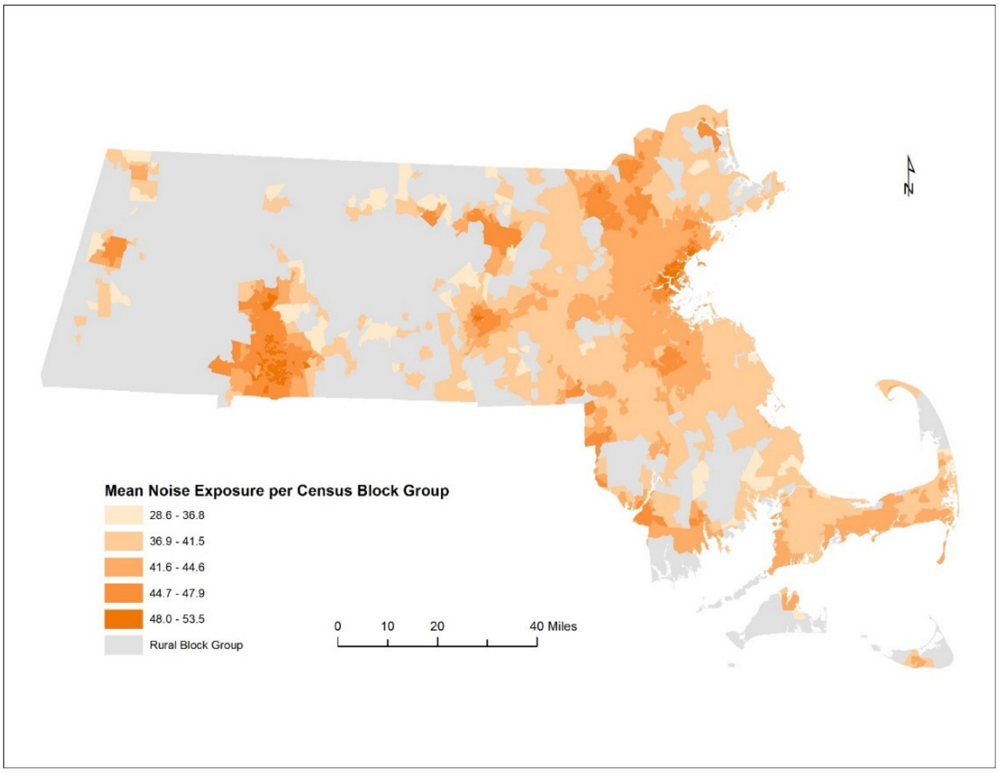

Figure S5. The mean noise exposure during pregnancy per census block group, among women who gave birth in Massachusetts between 2001 and 2011.

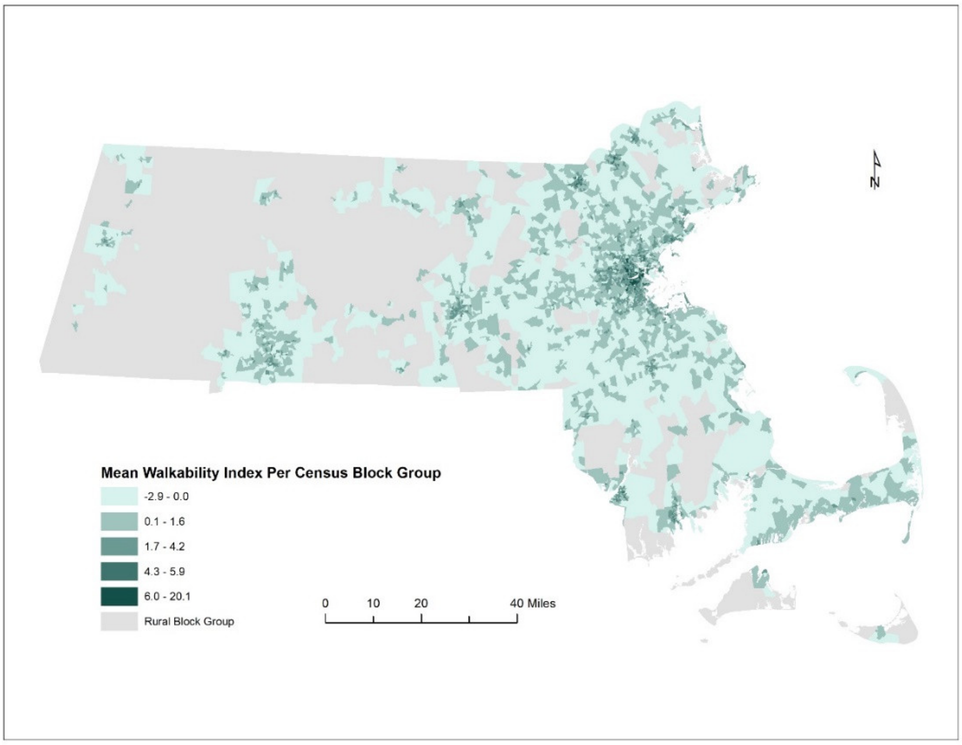

Figure S6. The mean walkability index during pregnancy per census block group, among women who gave birth in Massachusetts between 2001 and 2011.

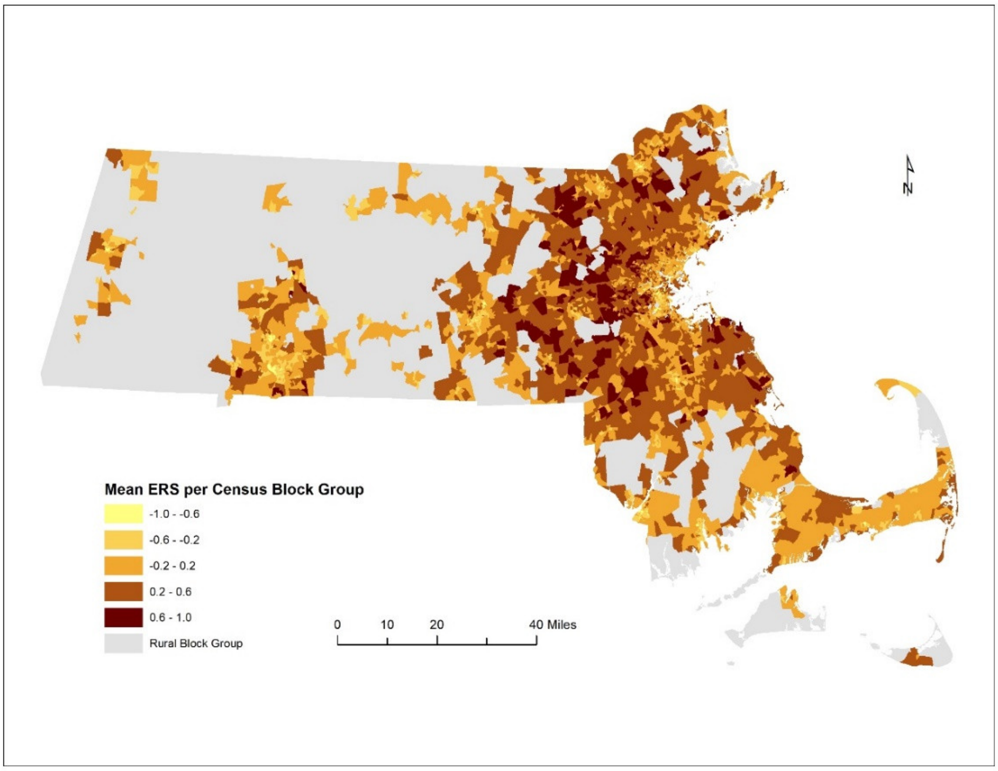

Figure S7. The mean economic residential segregation (ERS) exposure during pregnancy per census block group, among women who gave birth in Massachusetts between 2001 and 2011.

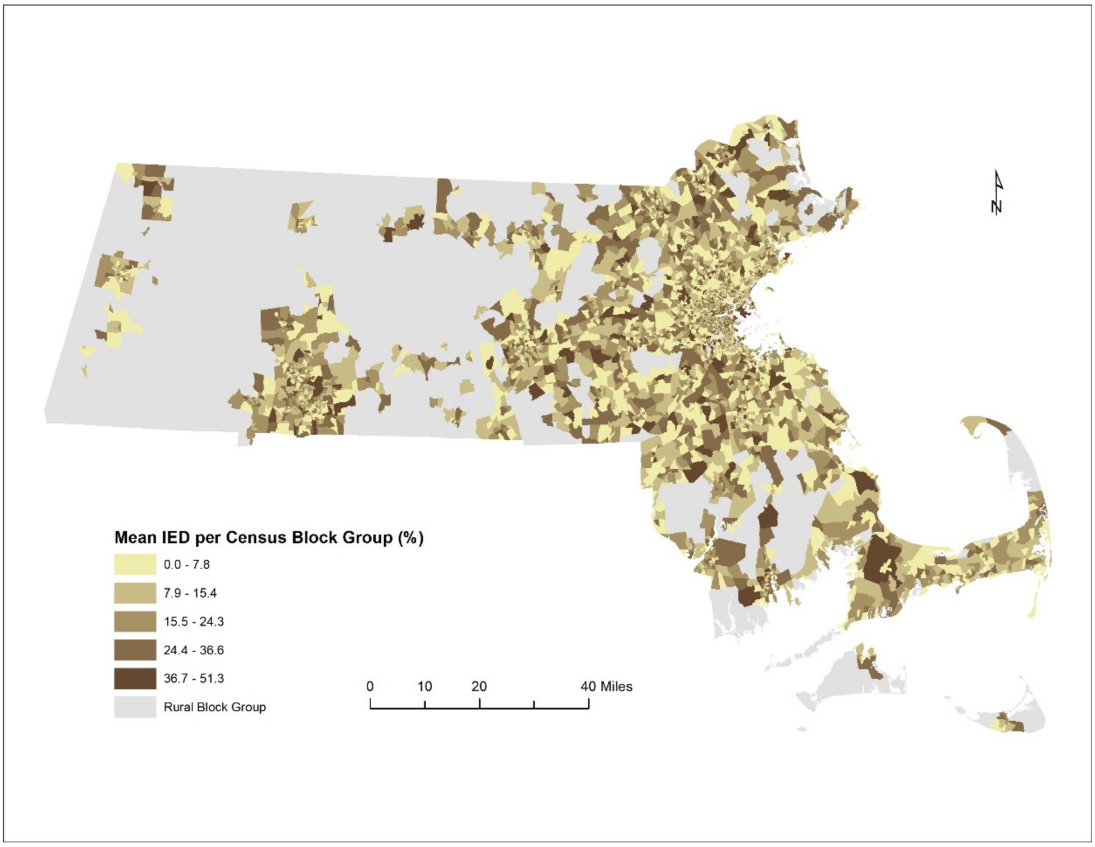

Figure S8. The mean index of economic dissimilarity (IED) exposure during pregnancy per census block group, among women who gave birth in Massachusetts between 2001 and 2011.

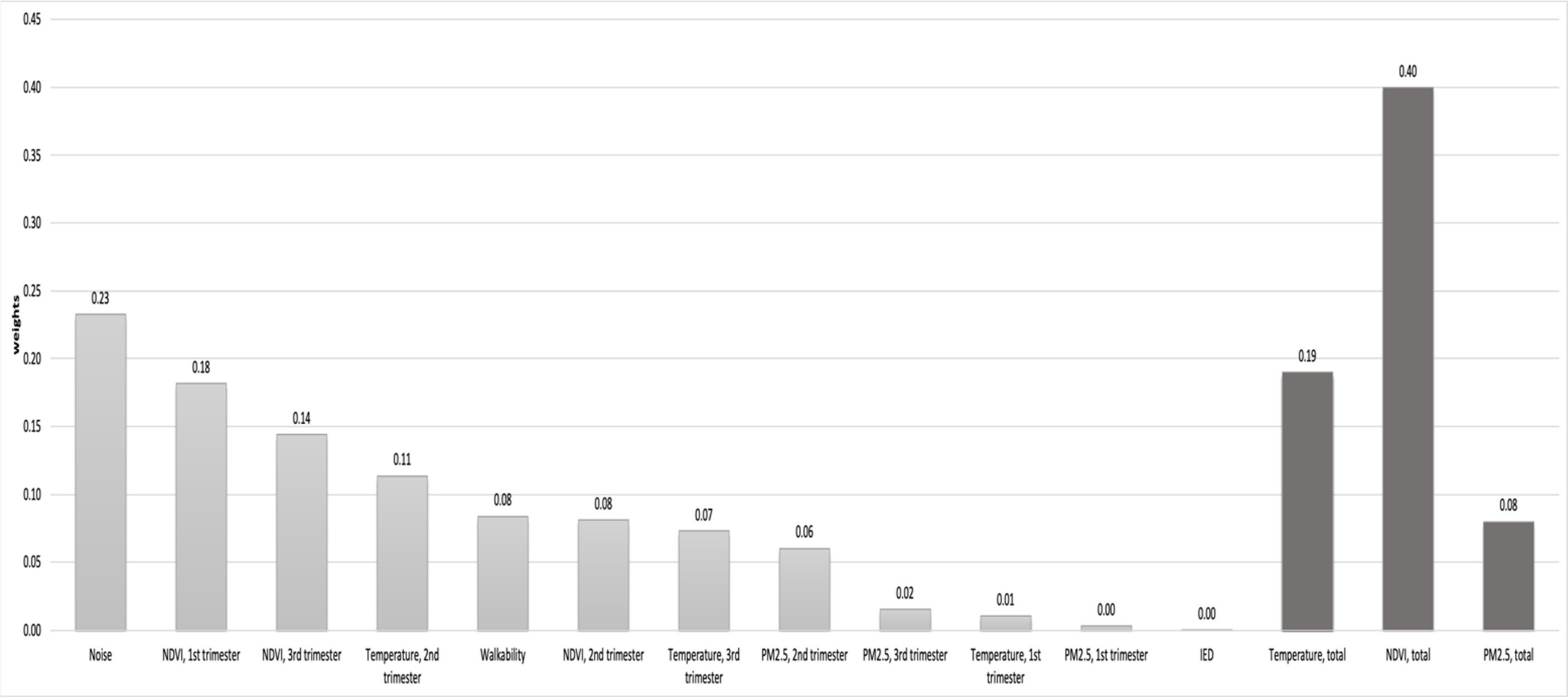

Figure S9. The weighted contribution of each of the selected exposures to a negative difference in birthweight: results of a Weighted Quantile Sum (WQS) regression with weights assigned to tertile-scored exposures.

Figure S9 shows the weighted contribution of each exposure to a negative difference in birthweight. Results were obtained from a WQS regression. The WQS regression summarizes all the exposures into one index while taking the association with the outcome into account, and the contribution of each exposure is weighted based on its relevance to the overall association with the outcome. The weights were assigned to tertile-scored exposures within the composite index and were constrained to be between zero and one and summing up to one.

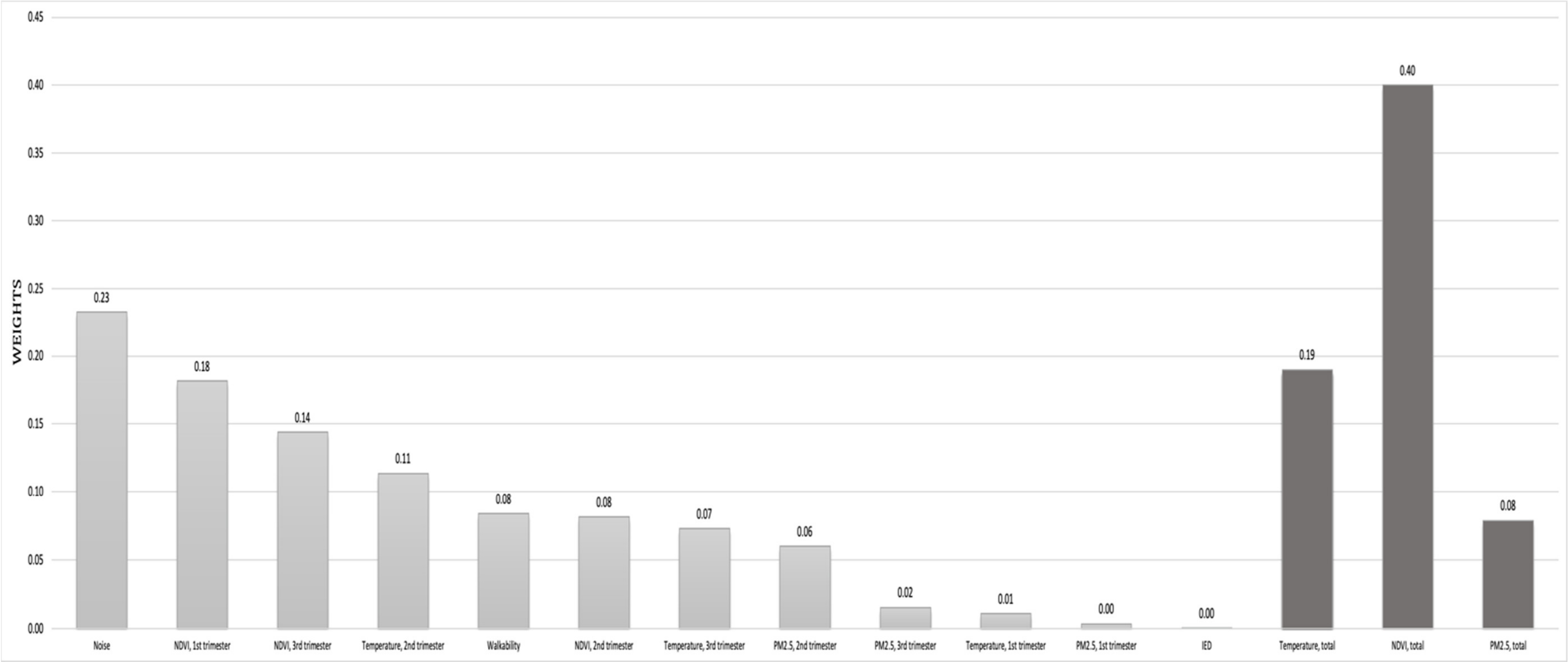

Figure 10. The weighted contribution of each of the selected exposures to a negative difference in birthweight: results of a Weighted Quantile Sum (WQS) regression with weights assigned to quintile-scored exposures.

Figure S10 shows the weighted contribution of each exposure to a negative difference in birthweight. Results were obtained from a WQS regression. The WQS regression summarizes all the exposures into one index while taking the association with the outcome into account, and the contribution of each exposure is weighted based on its relevance to the overall association with the outcome. The weights were assigned to quintile-scored exposures within the composite index and were constrained to be between zero and one and summing up to one.
